# Membranous nephropathy associated with hepatitis C virus infection treated with corticosteroids and Ledipasvir-Sofosbuvir: a case report and review of literature

**DOI:** 10.18632/oncotarget.15397

**Published:** 2017-02-16

**Authors:** Qinjie Weng, Xiao Li, Hong Ren, Jingyuan Xie, Xiaoxia Pan, Jing Xu, Nan Chen

**Affiliations:** ^1^ Department of Nephrology, Ruijin Hospital, Shanghai Jiaotong University School of Medicine, Shanghai, China

**Keywords:** hepatitis C virus, membranous nephropathy, hepatitis C virus-related glomerulonephritis, Ledipasvir-Sofosbuvir, case report

## Abstract

**Background:**

Membranous nephropathy (MN) is the most common cause of nephrotic syndrome in adults. As many clinical cases have reported, it may be associated with hepatitis C virus (HCV) infection. Antiviral therapy can be various.

**Case summary:**

We report a case of patient with chronic HCV infection and MN, who presented with was proteinuria. He was treated with ledipasvir and sofosbuvir (Harvoni; Gilead Sciences, Foster City, CA) and was found to be virus-free.

**Conclusion:**

We have reported this case to provide insight into whether Ledipasvir-Sofosbuvir should be administered for HCV-related glomerulonephritis.

## INTRODUCTION

Hepatitis C virus (HCV) infection is an important cause of chronic liver disease. Besides liver function impairment, it can lead to extrahepatic manifestations including kidney disease, such as membranous nephropathy (MN) [1–3].

Treatment of HCV-related MN is various, but no recommendation is provided. Antiviral therapy, which includes Interferon-α (INF-α) and ribavirin, is effective in clearing HCV infection in some patients [3–5]. In 2015, a new oral regimen is available for HCV patients. Ledipasvir-Sofosbuvir (Harvoni; Gilead Sciences, Foster City, CA) is a combination tablet and is recommended for patients with genotype 1[6, 7].

Here, we report a case of membranous nephropathy associated with HCV infection treated with ledipasvir and sofosbuvir, corticosteroid, and cytotoxic agent.

## CASE REPORT

A 65-year-old male patient was found to have proteinuria during a health checkup in 2014. He underwent a surgery for encephalic angioma and received massive blood transfusion in 1992. Besides, he had received a diagnosis of chronic HCV infection for 10 years. He was admitted to another hospital at first in January, 2015. Physical examination showed trace pitting edema of the lower extremities. The initial laboratory evaluation was significant for proteinuria of 2980mg/24h, serum albumin of 21g/L and HCV RNA viral load of 6.53*10^5 (copies). His HCV genotype was 1b. A renal biopsy was performed there, with histopathology consistent with membranous nephropathy. Light microscopy showed 15 glomeruli and one was global sclerotic. Thickened glomerular capillary walls were found. Tubulointerstitial lesion was mild. Immunofluorescence microscopy revealed diffuse granular capillary wall deposits of IgG [IgG1 (++), IgG2 (-), IgG3 (±), IgG4 (+++)], IgM, C3. They recommended to treat HCV infection first.

From April 10, the patient was started on Ledipasvir-Sofosbuvir (1#, qd) for 3 months. In the end of April, the patient presented to our hospital with progressive foamy urine. On admission, the patient's consciousness was clear. Physical examination showed severe edema of lower extremities. Blood pressure was 143/76mmHg and no clinical chest or abdominal abnormalities were found. Laboratory investigation revealed the following: serum creatinine 121μmol/L, eGFR_CKD-EPI_ 54ml/min/1.73m^2^, proteinuria 12169mg/24h, serum albumin 13g/L. Urinalysis was remarkable for protein (4+) and 31-50 red blood cells/high-power field (HPF). Complement factors, such as C3, C4, serum immunoglobulins and rheumatoid factor were in the normal range. Anti-dsDNA antibodies, anti-nuclear antibodies, and antineutrophil cytoplasmic antibodies were absent. HCV RNA viral load (Roche, COBAS AmpliPrep/COBAS TaqMan HCV Test) was already negative. Main laboratory findings were summarized in Table 1. Chest CT revealed a small amount of pleural effusion in the left side.

**Table 1:** Laboratory data of the patient

	Biopsy	3 months	7 months	12 months	20 months
Creatinine (μmol/L)	94	119	84	90	84
Proteinuria (mg/24h)	2980	12169	2258	1346	763
Albumin (g/L)	21	13	26	36	41
Hemoglobin (g/L)	144	134	110	129	124
Urinalysis	/	Protein (4+)	Protein (3+)	Protein (4+)	Protein (2+)
HCV RNA viral load (copies)	6.53*10^5	Negative	/	Negative	/
Autoimmunity	Normal	Normal	Normal	Normal	/

**Figure 1:**
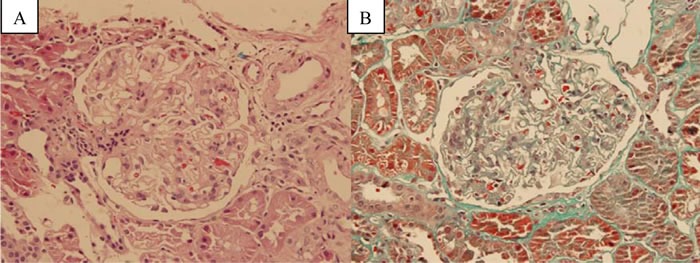
Kidney biopsy with light microscopy **A**. hematoxylin-eosin stain (×400). **B.** Masson stain (×400).

His pathology slides were read again: light microscopy revealed a total of 16 glomeruli, 2 of which were global sclerotic. Suspicious eosinophilic deposits were found in epithelial side in Masson staining. A diffuse thickening of glomerular basement membrane was seen with increased mesangial matrix and mesangial cells (Figure 1). Tubulointerstitial lesion was mild. These features were consistent with MN. Besides, HCV antibody was found to be negative in kidney tissues.

**Figure 2:**
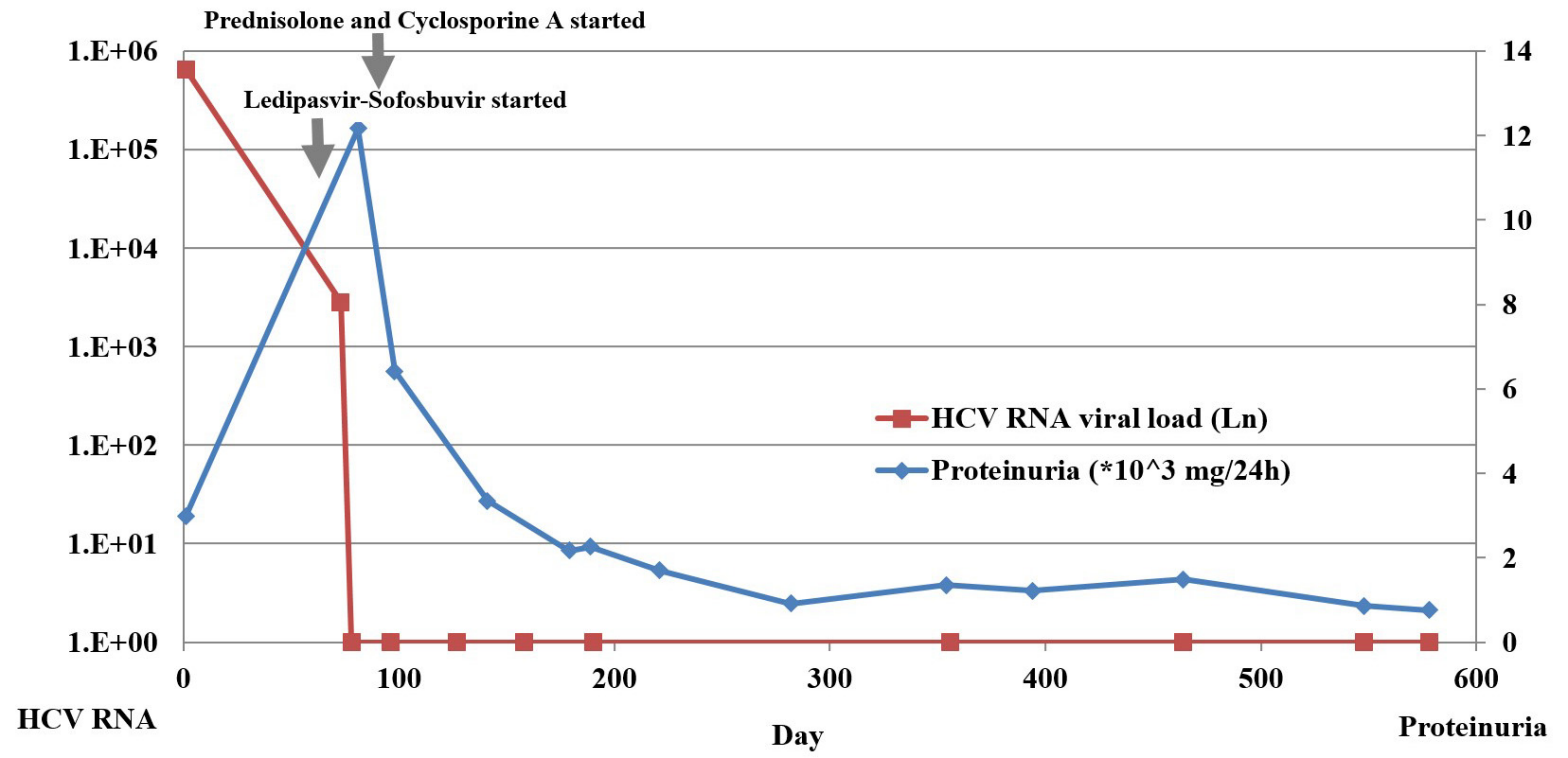
HCV RNA viral load and proteinuria at disease onset and during follow-ups

Steroid pulse therapy (40mg/d of methylprednisolone intravenously) was initiated for 3 days, then tapered to 40 mg/d of prednisolone orally with 200mg/d of Cyclosporine A. By then, he had been on Ledipasvir-Sofosbuvir for about 3 weeks. Also, he was treated with urokinase (50000U/d) for anticoagulation, Plavix for antiplatelet aggregation, along with Caltrate D, Rabeprazole, etc. During follow-ups, HCV RNA (COBAS) remained negative, meanwhile, serum creatinine decreased to normal (84μmol/L), proteinuria gradually decreased to 763mg/24h, and serum albumin increased to 41g/L, thus prednisolone and Cyclosporine A were gradually reduced. (Figure 2)

## DISCUSSION

This is a case of membranous nephropathy associated with HCV infection. The patient was treated with Ledipasvir-Sofosbuvir, followed with prednisolone and Cyclosporine A. To our knowledge, no such case was reported before.

The incidence of HCV infection has been reported to be about 3-4 million per year and it is known that 3% of the world's population—210 million people—is currently infected with HCV [4]. Meanwhile, HCV prevalence in the Chinese population was 1.6%. Annual incidence of HCV in China was 6.01 per 100,000 [8]. Almost 40% of patients with HCV develop at least one extrahepatic manifestation during the course of the disease, including kidney disease [9]. The prevalence of HCV infection was higher in hemodialysis patients than that in the general population of China [10].

Although Membranoproliferative glomerulo-nephritis (MPGN) is most commonly associated with HCV infection [11], other glomerulonephritis are also reported to be associated with HCV, including MN, focal segmental glomerulosclerosis (FSGS), postinfectious glomerulonephritis, IgA nephropathy, and fibrillary or immunotactoid glomerulopathy [12]. Yamabe et al [13] found that 8.3% of MN patients were anti-HCV-positive or HCV RNA-positive. The pathogenesis of MN in these patients may be related to the deposition of immune complexes containing HCV proteins in glomeruli, like HBV infection [14]. The clinical manifestation and histological findings of HCV-associated MN are similar to that of idiopathic MN.

The optimal therapy for HCV-associated glomerulonephritis is still not very clear [3, 15, 16]. The evidence on treatment of HCV-related kidney disease comes predominantly from IFN-based therapies [5]. By literature review, we found two case reports of patients with HCV-associated glomerulonephritis, presented with deterioration of kidney function and proteinuria.[17, 18] Treatment with INF-2a (one plus ribavirin) resulted in sustained virological response with a clinical remission of proteinuria as well as stabilization of renal function. Thus, we suggest that anti-virus therapy could ameliorate proteinuria in patients with HCV related glomerulonephritis.

Antiviral therapy, aiming for eradication and reduction of HCV-related antibodies and immune complexes, includes Interferon-α (IFN-α), Ribavirin, etc. It remains a main therapy in HCV-related glomerulonephritis [15]. The main treatment goal for HCV-infection is to achieve an undetectable HCV RNA level, which is known as sustained virological response (SVR), and is usually measured 12 weeks after completion of therapy [19]. Achieving SVR is considered a virological cure, as more than 99% of patients remain free from the virus when followed up for 5 years after completing therapy [19]. In the meta-analysis by Feng et al [20], antiviral treatment was found to decrease proteinuria in HCV-positive CKD patients. That is, the decrease in proteinuria following antiviral therapy was associated with HCV-RNA clearance.

Ledipasvir-Sofosbuvir is one of the newly discovered antiviral therapy, which was approved by the FDA on October 10, 2014, for the treatment of HCV infection. It is a novel, once-daily, fixed-dose combination antiviral agent that has shown high SVR rates in HCV-infected patients [6]. It contains both ledipasvir (an HCV NS5A inhibitor) and sofosbuvir (an NS5B polymerase inhibitor) and is the first anti-HCV therapy that does not require the coadministration of interferon or ribavirin [6, 7, 21].

Ledipasvir and sofosbuvir are both direct-acting antiviral agents [22]. Sofosbuvir is a liver-targeted nucleotide prodrug of the active triphosphate GS-461203, which has been approved for use in HCV genotypes 1-4. It acts as an inhibitor of the HCV NS5B RNA-dependent RNA polymerase, which works like a chain terminator [23, 24]. Ledipasvir is an NS5A inhibitor that is effective in genotypes 1a, 1b, 4a and 5a, and is also effective in genotypes 2a and 3a with relatively lower activity. The mechanism is still unclear, but one possible mechanism is that it inhibits hyperphosphorylation of NS5A, which is required for viral production. NS5A inhibitors may also lead to faulty viral assembly by redistributing the subcellular localization of the protein. The NS5A and NS5B inhibitors demonstrate an additive effect when used in combination [22].

Several studies about Ledipasvir-Sofosbuvir were carried out recently, all of which showed positive results. Afdhal [25] found that once-daily of Ledipasvir-Sofosbuvir with or without RBV for 12 or 24 weeks was highly effective in patients with HCV genotype 1 infection who are treatment naïve. Kowdley [26] concluded that once-daily of Ledipasvir-Sofosbuvir for 8 weeks was associated with a high rate of SVR among treatment naïve patients with HCV genotype 1 infection without cirrhosis. Meanwhile, they found that the inclusion of ribavirin in the regimen and the extension of the duration of treatment to 12 weeks were not associated with additional benefit. The study of Gane [27] in New Zealand showed that the combination of ledipasvir and sofosbuvir is highly effective in patients with HCV genotype 1 infection who are treatment naïve or did not respond to previous treatment.

The most common adverse events observed in clinical trials [25, 26, 28] with Ledipasvir-Sofosbuvir were headache, fatigue, nausea, and insomnia. Hematologic abnormalities also occurred in some patients, including mild-to-moderate hyperbilirubinemia, transient and asymptomatic lipase elevations, and a mean hemoglobin change of -0.2 g/dL to -0.6 g/dL from baseline. Maximum plasma concentrations (Tmax) of ledipasvir were observed 4 to 4.5 hours post oral administration with a terminal half-life (t1/2) of 47 h. Sofosbuvir undergoes intracellular activation and ultimately becomes GS-331007, a predominant circulating metabolite, which has peak plasma concentrations of 3.5 to 4 hours and a half-life of 27 h [29]. No obvious nephrotoxicity was observed in patients using Ledipasvir-Sofosbuvir. However, we do find a case report [30] of Ledipasvir-Sofosbuvir associated biopsy-proven acute interstitial nephritis (AIN). As Ledipasvir is eliminated in the feces, whereas the majority of sofosbuvir is cleared by the kidney, patients with CKD who have impaired excretion may experience a prolonged exposure of its metabolites and delayed recovery of renal function [31]. Sofosbuvir-containing regimens are not currently recommended for patients with estimated Glomerular Filtration Rate (eGFR) less than 30 ml/min. Currently, dose recommendations cannot be given for patients with advanced renal disease [32]. Further studies in larger cohorts and improvements in safety and efficacy are needed for patients with renal disease. Therefore, physicians need to be aware of the potential side effect of this agent.

Regarding to the present case, this is a patient with chronic HCV infection and nephrotic syndrome. It is usually difficult to design treatment for these patients since steroids can exacerbate HCV infection and lead to inferior results. As can be seen in this case, antiviral therapy (Ledipasvir-Sofosbuvir) followed by steroids and cytotoxic agent can not only reduce urinary protein, improve hypoproteinemia, relieve edema, but also effectively prevent virus reactivation and further aggravation of HCV infection. Therefore, in this case, Ledipasvir-Sofosbuvir shows positive effect and ensures the safety of the following steroids treatment. However, due to the side effects of steroids and antiviral therapy, physicians should still be cautious with the treatment in case of any complication.

## CONCLUSIONS

We have reported a case of membranous nephropathy with HCV infection. The patient was treated with Ledipasvir-Sofosbuvir for antiviral therapy, along with prednisolone and Cyclosporine A. He remained free from virus and his proteinuria was gradually reduced during follow-up for 20 months even after stopping Ledipasvir-Sofosbuvir. We believe this case report provides some insight into whether Ledipasvir-Sofosbuvir should be administered for glomerulonephritis with HCV infection.
